# A Literature Review of Similarities Between and Among Patients With Autism Spectrum Disorder and Epilepsy

**DOI:** 10.7759/cureus.33946

**Published:** 2023-01-18

**Authors:** Freda B Assuah, Bryce Emanuel, Brianna M Lacasse, John Beggs, Jennie Lou, Francis C Motta, Louis R Nemzer, Robert Worth, Gary D Cravens

**Affiliations:** 1 Osteopathic Medicine, Nova Southeastern University Dr. Kiran C. Patel College of Osteopathic Medicine, Clearwater, USA; 2 Halmos College of Arts and Sciences, Nova Southeastern University, Fort Lauderdale, USA; 3 Physics, Indiana University, Bloomington, USA; 4 General Internal Medicine, Wake Forest School of Medicine, Winston-Salem, USA; 5 Mathematical Sciences, Florida Atlantic University, Boca Raton, USA; 6 Mathematical Sciences, Indiana University School of Medicine, Indianapolis, USA; 7 Health Informatics, Nova Southeastern University Dr. Kiran C. Patel College of Osteopathic Medicine, Fort Lauderdale, USA

**Keywords:** epileptic seizures, neurological disorder, co-occurring epilepsy and autism, epilepsy comorbidity, autism comorbidity, asd and epilepsy, asd, autism, epilepsy, autism spectrum disorder (asd)

## Abstract

Autism spectrum disorder (ASD) has been shown to be associated with various other conditions, and most commonly, ASD has been demonstrated to be linked to epilepsy. ASD and epilepsy have been observed to exhibit high rates of comorbidity, even when compared to the co-occurrence of other disorders with similar pathologies. At present, nearly one-half of the individuals diagnosed with ASD also have been diagnosed with comorbid epilepsy. Research suggests that both conditions likely share similarities in their underlying disease pathophysiology, possibly associated with disturbances in the central nervous system (CNS), and may be linked to an imbalance between excitation and inhibition in the brain. Meanwhile, it remains unclear whether one condition is the consequence of the other, as the pathologies of both disorders are commonly linked to many different underlying signal transduction mechanisms. In this review, we aim to investigate the co-occurrence of ASD and epilepsy, with the intent of gaining insights into the similarities in pathophysiology that both conditions present with. Elucidating the underlying disease pathophysiology as a result of both disorders could lead to a better understanding of the underlying mechanism of disease activity that drives co-occurrence, as well as provide insight into the underlying mechanisms of each condition individually.

## Introduction and background

Autism spectrum disorder (ASD) is a neurodevelopmental disorder marked by impairments in social phenotypes and communication, as well as repetitive and restricted behaviors. The World Health Organization (WHO) currently reports a prevalence of 1 in every 100 children, while the Centers for Disease Control and Prevention (CDC) reports a prevalence of 2 per 100 eight-year-olds in the United States [1,2]. This age limit was selected because most children with ASD have been identified by that age. ASD can be attributed to a variety of risk factors, including biological, genetic, and environmental factors [2]. ASD diagnosis usually begins with the screening of children in pediatric care and concludes with a further diagnosis from a clinician with expertise in ASD. Ongoing research efforts aim to understand the etiology tied to ASD, as there are currently no defined etiologies, solely risk factors [3].

Meanwhile, epilepsy is a neurological disorder, defined by a propensity for recurrent seizures [4]. The WHO reported that the prevalence of epilepsy averages approximately 50 million individuals globally [5]. Currently, the occurrence of epilepsy in the United States is approximately 3.4 million individuals, and the etiologies for epilepsy vary widely [6]. Epilepsy can be caused by a variety of factors, which the WHO categorizes into structural, genetic, infectious, metabolic, immune, and unknown [5]. Epilepsy can be managed well with antiepileptic medication, but there is a subset of patients who can be refractory to currently available treatment options [5]. Current challenges in the effective management and targeted treatment of epilepsy can be attributed to limited data on the underlying mechanisms of seizure onset and progression [6].

The co-occurrence of ASD and epilepsy has been recorded to be a significant prevalence. In a systematic review conducted with studies reporting 283,549 patients, the authors found a 12.1% median overall prevalence of epilepsy in individuals with ASD [7]. The percentage is significant compared with the 1.03% of the United States population with epilepsy [6]. While research efforts demonstrate that there is likely an underlying pathological mechanism of disease activity linked between the two conditions, this link is yet to be fully elucidated. Research suggests that both conditions likely share similarities in their underlying disease pathophysiology, likely associated with disturbances in the central nervous system (CNS), and may be linked to an imbalance between excitation and inhibition in the brain. Specifically, researchers have demonstrated the association of both conditions with disturbances in the CNS may be tied to defects of gamma-aminobutyric acid (GABA-A) neuron receptors or immune dysregulation leading to neuron damage [8]. Other researchers hypothesize that the aberrant activation of the mammalian target of rapamycin (mTOR) is associated with both ASD and epilepsy [9]. The condition, tuberous sclerosis complex (TSC), which affects the mTOR pathway, often has ASD and epilepsy as comorbidities [10]. Still, it remains unclear whether one condition can be the consequence of the other, as the pathologies of both disorders are commonly associated with many different underlying signal transduction mechanisms. In this review, we aim to investigate the comorbidity of ASD and epilepsy, with the intent of gaining insights into the similarities in pathophysiology that both conditions present with.

## Review

Method

Literature Search

**Figure 1:**
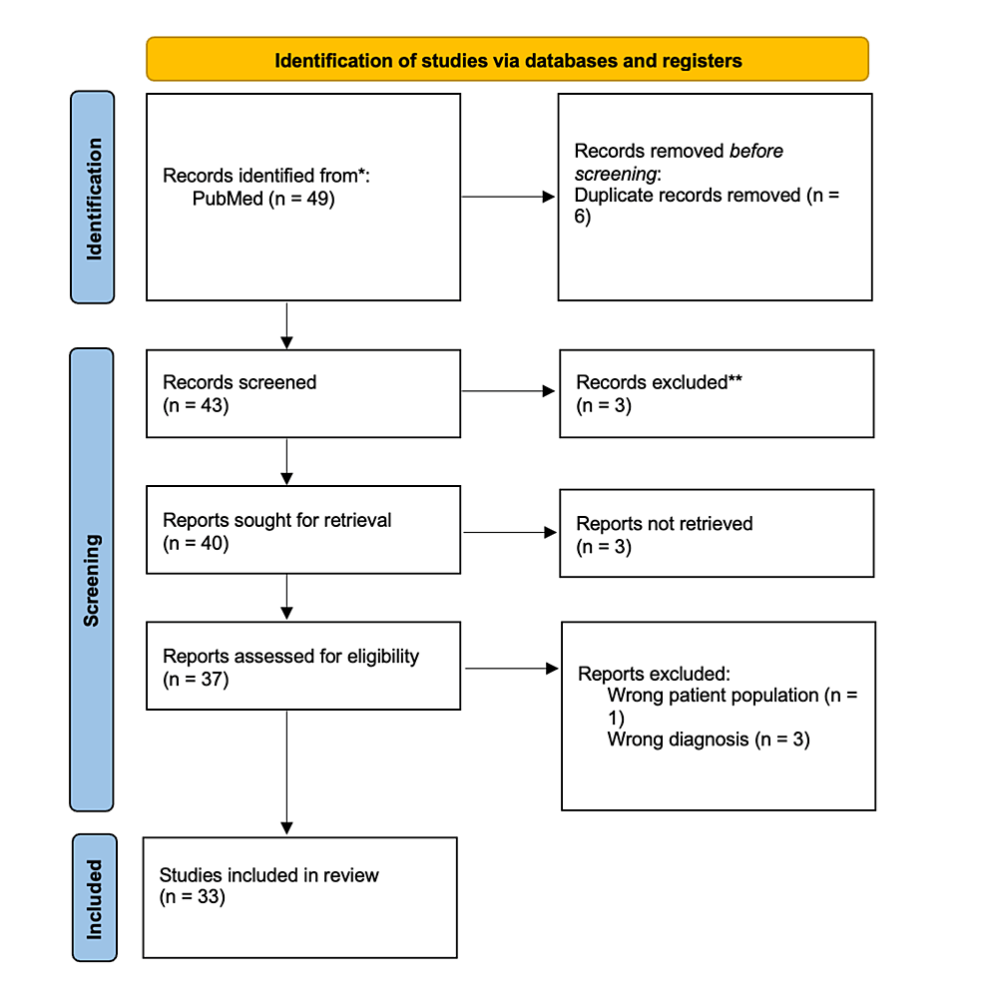
PRISMA flow diagram for literature review search. The first, second, and third authors created the image using the following template from: Page MJ, McKenzie JE, Bossuyt PM, Boutron I, Hoffmann TC, Mulrow CD, et al. The PRISMA 2020 statement: an updated guideline for reporting systematic reviews. BMJ 2021;372:n71. doi: 10.1136/bmj.n71 http://www.prisma-statement.org/ PRISMA, Preferred Reporting Items for Systematic Reviews and Meta-Analyses

Our literature search was done via PubMed, with the following search criteria for all the keywords: (1) articles from January 01, 2012, to March 31, 2022; (2) free full-text availability; (3) English text; (4) article categories included randomized controlled trial, clinical trial, systematic review, review, and meta-analysis; and (5) human trials only. Three queries were run. The first was the keyword “Autism,” which yielded 3,144 results; when Boolean AND with the keyword “comorbidity” was applied, there were 224 results; and when Boolean AND with the keyword “epilepsy” was then applied, this yielded 28 results. The second query using the previously stated search criteria was run with the keyword “epilepsy,” which yielded 4,258 results. When Boolean AND with the keyword “co-occurring Autism” was applied, it yielded 13 results. The third query was run with the keywords “autism clinical manifestation,” which yielded 79 results. These results were further narrowed down with Boolean AND with the keyword “epilepsy” to yield eight results. At the end of all the queries, there were a total of 49 articles. Duplicates as well as articles that did not explicitly discuss ASD or epilepsy or possible etiologies, either in their abstracts or in their titles, were removed. The total number of articles at the end of this initial analysis was 33, which were included in this study. The search process and article selection are illustrated by the Preferred Reporting Items for Systematic Reviews and Meta-Analyses (PRISMA) diagram in Figure 1.

Results

Among the 33 articles included in this literature review, the strength of the articles was categorized based on the type of article or studies completed. We categorized articles that were randomized controlled trials, systematic reviews, and meta-analyses as very strong and review articles as strong. The articles that indicated various co-occurrence statistics and similarities are summarized in Table 1.

**Table 1:** Summary of citations for literature used. ASD, autism spectrum disorder; ID, intellectual disability; TSC, tuberous sclerosis complex; IDD, intellectual developmental disability; IDD-E, intellectual developmental disability and epilepsy; mTOR, mammalian target of rapamycin; EFMR, epilepsy and mental retardation limited to females; DNA, deoxyribonucleic acid; GABA, gamma-aminobutyric acid; IL, interleukin

Article	Co-occurrence	Biological similarities	Type of study	Strength of study
Agarwal et al. [11]	Epilepsy is listed as a common symptom of autism.	The synthetic modulation of the endocannabinoid system is associated with the treatment of ASD and epilepsy through the use of cannabis.	Meta-analysis	Very strong
Baasch et al. [12]		Gene mutations such as SCN2A mutations are found in patients with ID and autism.	Randomized controlled trial	Very strong
Bădescu et al. [13]		Microdeletions in the long arm of chromosome 21, specifically region q21.1, increase the risk of developing autism and epilepsy.	Review article	Strong
Buckley and Holmes [14]	The prevalence of epilepsy in ASD can vary and was reported as almost 50%.	TSC and Fragile X syndrome are two conditions that show a high incidence of both ASD and epilepsy.	Review article	Strong
Carroll et al. [15]	ASD has the comorbidity of epilepsy listed in this article.	The protein CNTNAP2 mutation is associated with both ASD and epilepsy.	Review article	Strong
Catterall et al. [16]		Dysfunction in sodium channels is associated with Dravet syndrome, an epilepsy disorder with comorbidity of autism.	Review article	Strong
Chahrour et al. [17]	ASD is associated with epilepsy as a comorbidity in this article.		Review article	Strong
Devinsky et al. [18]	18% of people with IDD were reported as having epilepsy.	Deletions and duplications in chromosomes 15 and 16 are found in people with IDD-E.	Review article	Strong
Frye et al. [19]	The prevalence of epilepsy in ASD is reported from 5% to 39% compared to 1% to 2% in the general childhood population.	Epilepsy occurring in children with ASD is a common symptom of an underlying metabolic disorder in children who present with both.	Review article	Strong
Guerrini and Parrini [20]		Alterations in the FOXG1 gene have been associated with clinical features such as epilepsy and autism.	Review article	Strong
Hester and Danzer [21]	Temporal lobe epilepsy can be associated with autism.	mTOR signaling pathway appears to be associated with the development of autism and epilepsy.	Review article	Strong
Holmes [22]	Epilepsy is commonly associated with ASD with a prevalence of almost 50%.	Some antiseizure medications such as cannabidiol can help treat anxiety associated with ASD.	Review article	Strong
Howes et al. [23]	Co-occurring conditions such as epilepsy are common in ASD.	The excitatory and inhibitory model associated with ASD, although simplistic, can also be associated with epilepsy.	Review article	Strong
Jacob [24]	In 30% of ASD subjects, epilepsy can be observed as a comorbid condition.	The cortical interneuron circuit has been found as one important mediator in ASD and epilepsy.	Review article	Strong
Jeste and Tuchman [25]	Epilepsy has been reported at a prevalence of 6% to 27% in individuals with ASD.	The two theories regarding the pathophysiology of epilepsy and ASD are the excitatory/inhibitory model and genetic mutations.	Review article	Strong
Jensen [4]		Epilepsy and autism are assumed to be caused by a primary disruption of synaptic function or genetic mutation.	Research article	Strong
Kolc et al. [26]	People with more severe intellectual disabilities are often found to have seizure onset within the first 12 months of life.	The disorder, EFMR can be attributed to mutations in the X-chromosome gene PCDH19.	Review article	Strong
Lee et al. [27]	ASD and epilepsy’s co-occurrence are well recognized.	The different biological pathways involved in both ASD and epilepsy include gene transcription regulation, cellular growth, synaptic channel function, and maintenance of the synaptic structure.	Systematic review/Meta-analysis	Very strong
Leuzzi et al. [28]		The guanidinoacetate methyltransferase deficiency presents with epilepsy and autistic-like behavior.	Review article	Strong
Mizuguchi et al. [29]	Tuberous sclerosis complex is commonly associated with epilepsy and ASD.	Treatment with everolimus leads to improvement in symptoms of ASD and epilepsy.	Randomized controlled trial	Very strong
Mühlebner et al. [9]	Malformations in the mTOR signaling pathway are linked to different developmental malformations such as epilepsy and autism.	The malformations in the mTOR pathways are linked to germline and somatic mutations.	Review article	Strong
Neymotin and Nemzer [8]	There are 15% to 30% of children with epilepsy who also have ASD.	Malfunctioning of gamma-aminobutyric acid-A neuron receptors can lead to both ASD and epilepsy.	Review article	Strong
Noebels [30]	Nearly half of the clinical autism features of a study of patients with childhood-onset epilepsy arose after the onset of their seizures.	The genetic mutations that affect neurotransmission and DNA methylation and chromatin remodeling are overlapping biological features of autism and epilepsy.	Review article	Very strong
Pan et al. [31]	Epilepsy is found to be more common in people with autism than by chance, with a prevalence of 11.3% to 17.2%.		Systematic review	Very strong
Prager et al. [32]		Alterations in the GABAergic system are linked to both autism and epilepsy.	Review article	Strong
Rybakowski et al. [33]	Epilepsy is found in about 25% of children with autism.	Excitatory mechanisms in the cerebral cortex more than inhibitory mechanisms are found to be linked with both epilepsy and autism.	Review article	Strong
Strasser et al. [34]	In individuals with ASD, the lifetime prevalence of developing epilepsy can be as high as 44.4%.	Given that syndromic ASD is found to have more prevalence of the occurrence of epilepsy, it is hypothesized that an underlying neurological condition is responsible for both conditions.	Systematic review	Very strong
Tee et al. [10]	People with TSC are often found to have epilepsy and autism.	Genetic mutations leading to activation of the mTOR pathway is found to be the underlying etiology in TSC co-occurring with epilepsy and autism.	Review article	Strong
Vasic et al. [35]		Variations in the mTOR pathway genes are found to be associated with ASD and strongly comorbid with epilepsy.	Review article	Strong
Velíšková et al. [36]	Epilepsy and seizures are commonly seen in patients with ASD.	Changes in excitable networks caused by inflammatory regulators as well as brain network coordination contribute to both epilepsy and ASD.	Review article	Strong
Wei and Lee [37]	There is an estimated 20% to 25% of children with ASD who have an increased prevalence of seizures.	The co-occurrence of ASD with epilepsy suggests an underlying encephalopathy. The children also report having a younger age of onset of two years old.	Review article	Strong
Weldon et al. [38]		The loss-of-function mutation of the SYNGAP1 gene is a common cause of intellectual disability occurring with epilepsy.	Review article	Strong
Zahra et al. [39]	There is an apparent association between severe epileptic encephalopathy with ASD.	The downregulated expression of IL-6 and the upregulated expression of IL-12p40 combine to create a risk for epilepsy as a comorbidity of autism.	Systematic review	Very strong

Discussion

The prevalence of epilepsy and ASD co-occurring varied widely, with some studies reporting almost 50% in rates of comorbidity among patients [14]. In a meta-analysis carried out in 2012, of the 16 studies with a sample of patients with ASD, the rate of epilepsy was 8.9% in individuals without an intellectual disability (ID), but 23.7% in those with an ID [14]. In a large cross-sectional study covered in the same meta-analysis evaluating 5,815 individuals with ASD, the average prevalence of epilepsy differed by age of the cohort examined, with a prevalence rate of 12.5% in children aged between 2 and 17 years, but 26% in those aged 13 years or older [14]. Similarly, in a study of children in an English school system, there was a prevalence of ASD in children with epilepsy and 21% of the children had ASD [14}. Although, the percentages vary across the literature and examples cited, we found that the majority of the literature reviewed showed an increased co-occurrence of epilepsy and ASD.

While ASD has been previously posited as more common among males, a recent meta-analysis, which did not use the Diagnostic and Statistical Manual of Mental Disorders, Fifth Edition (DSM-5) criteria, found that the true male-to-female ratio is closer to 3:1 than the previously reported 4:1 [3]. According to this study, females with ASD were more likely to go without a clinical diagnosis of ASD as misdiagnosis, delayed diagnosis, or omission from treatment may be related to the female ASD phenotype, as many females do not exhibit observable symptoms [3]. In addition, females tend to camouflage their social deficits, which makes an early diagnosis difficult. Similarly, misconceptions about ASD being a male condition and gender biases could prevent diagnoses in females [3]. These findings emphasize the importance of not only addressing the comorbidity of ASD and epilepsy but also the way in which we approach this intersection, where biases such as sex may affect diagnostic approaches.

Gaining a better characterization of both ASD and epilepsy, occurring together and alone, is important, as this improved understanding could lead to improved diagnostic markers and more targeted therapeutic interventions. A randomized controlled trial using antiepileptic drugs (AEDs) such as everolimus, in patients with comorbid ASD and epilepsy, reported improvements in seizure activity as well as ASD symptoms based on their Pervasive Developmental Disorders-Autism Society Japan Rating Scale (PARS) score [29]. In a nonselected series study of 13 children undergoing treatment for infantile spasms due to tuberous sclerosis complex, with vigabatrin, ASD behavior disappeared in 5 out of the 6 children who were spasm free by the end of the study [40]. These treatments, although limited in their scope, support the concept that advancements in the treatment of epilepsy could contribute to the reduction of ASD manifestation as these conditions likely share multiple mechanisms of action.

The signal transduction mechanisms of both ASD and epilepsy have been found to lean more toward excitation than inhibition [24]. In addition, the cortical interneuron functions are found to be a core dysfunction in both ASD and epilepsy. These cortical interneurons express the inhibitory neurotransmitter, GABA, and the dysfunction of the interneurons further leads to excitation [24]. A potential correlation of epilepsy and ASD is that recurrent seizures could lead to permanent damage to cortical neuronal networks that control behavior [24]. The excitation of the signal transduction pathway is also linked to the tuberous sclerosis complex patients who often exhibit both ASD and epilepsy as comorbidities [10].

Research has suggested that correlations in genetic mutations may contribute individually to both ASD and epilepsy as well as in co-occurrence. A study on the mutation of the *SYNGAP1* gene leading to a loss of function is associated with a specific form of ID known as autosomal mental retardation type 5 (MRD5), which is associated with epilepsy as a symptom [38]. Therefore, the loss of function of the *SYNGAP1* gene has been reported as one of the most common causes of ID with epilepsy due to its prevalence in people with both epilepsy and ASD [38]. In addition, the deletion of the long arm of chromosome 21 in the chromosomal region q21.1 shows an increased risk for both ID and epilepsy [13]. It should be noted that although both describe the findings as similar in patients with both ASD and epilepsy, the genes are also related to other conditions, including hypotonia and other intellectual disorders.

**Figure 2:**
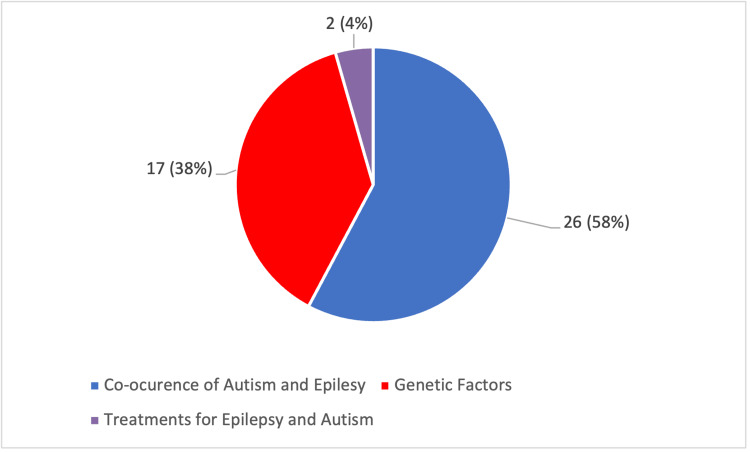
Pie chart for focus areas of literature used. The total number of articles is 45. The red section indicates articles that discussed genetic factors. The blue section indicates articles that discussed the co-occurrence of autism and epilepsy. The purple section indicates articles that discussed treatments for epilepsy and autism.

Figure 2 displays a pie chart exhibiting the number of articles that show the co-occurrence of ASD and epilepsy, those that mention genetic factors, and those that mention treatments for both.

Limitations

This review is not without limitations. Although epilepsy and autism may co-occur frequently, epilepsy has a wide range of etiologies. It is difficult to determine if ASD is one of the causes or if they share a similar pathophysiology [14]. The same can be said for ASD and its relationship with epilepsy [14].

## Conclusions

The primary aim of this study was to evaluate the co-occurrence of ASD and epilepsy and understand if similarities in biological mechanisms exist between ASD and epilepsy. In terms of the co-occurrence of ASD and epilepsy, based on our findings, epilepsy is more prevalent in individuals with ASD than in individuals without ASD. The co-occurrence between males and females is yet to be determined, with males predominately being more frequently diagnosed with ASD compared with their female counterparts. In terms of underlying biological mechanisms, the pathophysiology of the co-occurrence of both conditions, although not fully understood, is suggested to be associated with the overexcitation of the brain signal transduction, including defects of GABA-A neuron receptors or immune dysregulation leading to neuron damage as well as aberrant activation of the mTOR pathway. Correlations in genetic mutations may also contribute individually to both ASD and epilepsy as well as in their co-occurrence. Given the varying etiologies of epilepsy, it is difficult to ascertain the direct relation between ASD and epilepsy. While some epilepsy treatments such as vigabatrin and everolimus have been demonstrated to improve symptoms of ASD in patients with both comorbid ASD and epilepsy, it remains unclear whether the intersection between ASD and epilepsy could lead to the identification of other overlapping mechanisms and improve diagnosis and target treatment. High co-occurrence, similarities in biological mechanisms, treatment effectiveness, and genetic polymorphisms of ASD and epilepsy create a foundation for further research. Going forward, more research is needed to better characterize an understanding of the similarities and target diagnostic tools and treatment more effective for both disorders. In addition, a question to be further explored that was not answered in the scope of this study is if the co-occurrence of ASD and epilepsy can suggest a unique diagnosis. As further work is done on the relationship between the pathophysiology of both conditions, the treatment and clinical management of both conditions could be improved.
